# Supplementary material for: G‐quadruplex‐binding small molecules ameliorate C9orf72 FTD/ALS pathology in vitro and in vivo
Source: EMBO Mol Med. 2017 Nov 7;10(1):22–31. doi: 10.15252/emmm.201707850 (PMC5760849; doi:10.15252/emmm.201707850)
Supplement: Supplementary file 1 — Appendix [file EMMM-10-22-s001.pdf]

Appendix
Supplementary information

G-quadruplex-binding small molecules ameliorate *C9orf72* FTD/ALS pathology *in vitro* and *in vivo*

Roberto Simone, Rubika Balendra, Thomas G. Moens, Elisavet Preza, Katherine M. Wilson, Amanda Heslegrave, Nathan S. Woodling Teresa Niccoli, Javier Gilbert-Jaramillo, Samir Abdelkarim, Emma L. Clayton, Mica Clarke, Marie-Therese Konrad, Andrew J. Nicoll, Andrea Calvo, Adriano Chio, Henry Houlden, James M. Polke, Mohamed A. Ismail, Chad E. Stephens, Tam Vo, Abdelbasset A. Farahat, W. David Wilson, David W. Boykin, Henrik Zetterberg, Linda Partridge, Selina Wray, Gary Parkinson, Stephen Neidle, Rickie Patani, Pietro Fratta, and Adrian M. Isaacs.

Table of contents

	Page
[illegible] Supplementary Methods	2
[illegible] Supplementary Figures	5
[illegible] Supplementary Tables	15
[illegible] Supplementary References	16

Appendix supplementary methods

Fluorescence anisotropy titration

Florescence anisotropy was performed for DB1246 and DB1247 but was not possible for DB1273 due to its weaker fluorescence. Desalted 5'- rGrGrGrC rCrGrGrGrG rCrCrGrGrG rGrCrCrGrG rGrG -3' (G-Q-RNA) (IDT, Coralville, IA, USA) was suspended in 50 mM KCl, 10 mM Tris-HCl (pH 7.4) in nuclease-free water to a final concentration of 1 mM. Samples were heated to 98°C for 10 minutes, cooled for 45 minutes and stored at 4 ºC before use. Fluorescence polarization measurements were obtained on a Variant Cary Eclipse Fluorescence Spectrophotometer (Agilent Technologies, Santa Clara, CA) equipped with a polarization attachment. The excitation and emission polarizers were inserted between the light source and detector, respectively. Measurements were converted into anisotropy value by using Equations 1-2:

$$r = \frac{I_{vv} - GF * I_{vh}}{I_{vv} + 2 * GF * I_{vh}} \qquad (1)$$

$$GF = \frac{I_{hv}}{I_{hh}} \qquad (2)$$

Where r is the anisotropy value; I_{vv} is the fluorescence intensity of compound when excitation and emission polarizers are aligned in vertical position; I_{vh} is the fluorescence intensity of compound when the excitation and emission polarizer are in vertical and horizontal position, respectively; I_{hv} is the fluorescence intensity of compound when excitation and emission polarizer align in horizontal and vertical positions; I_{vv} is the fluorescence intensity of compound when excitation and emission polarizer align in vertical position; GF stands for the grating factor of the polarizers. GF values were introduced to the equation as a means to correct the unequal sensitivity of the excitation and emission polarizers (Lea *et al*, 2011). To determine equilibrium constants the compounds were added into a quartz fluorescence cuvette at a fixed concentration of 50 nM and G-Q-RNA was titrated into the cuvette. The amount of G-Q-RNA was calculated before each titration experiment and the concentration of fluorophore was corrected after each addition for the slight dilution. Measurements were taken at the compound maximum emission wavelength. Both excitation and emission slits were at 20 nm. Each set of measurements were taken from a 50 s average reading measurement. The experimental buffer conditions were 50 mM KCl, 10 mM Tris-HCl pH 7.3 and 1 mM EDTA. The dissociation constant (K_D) of DB compounds to G-Qs was obtained by fitting the change in anisotropy value of the compound titration by using the following equation for a 1 to 1 binding reaction:

$$r_{observed} = r_{min} + [(r_{max} - r_{min}) \frac{K_D + L + D - \sqrt{(K_D + L + D)^2 - 4LD}}{2L}] \qquad (3)$$

where r_{min} is the anisotropy value of the unbound molecule; r_{max} is the anisotropy value of fully bound molecule; L: total concentration of fluorophore; D: total concentration of receptor (G-Qs) (Roehrl *et al*, 2004)

Southern blotting

RNA free genomic DNA was extracted from cultured cells using the Qiagen Puregene kit following manufacturer's instructions. 5 µg of genomic DNA was digested using EcoRI and BamHI (NEB) and run on a 0.8% agarose gel at 60 V overnight. The gel was agitated for 30 min in 1L of denaturation buffer (National Diagnostics) followed by 1L of neutralisation buffer (National Diagnostics). DNA was transferred onto a positively charged nylon membrane (Roche Applied Science) via capillary action overnight using 20X SSC as transfer buffer. Following transfer, DNA was baked onto the nylon membrane at 80 ºC for 2 hours. Membrane was briefly wetted in water before being incubated in DIG Easy Hyb buffer

(Roche) at 46 ºC for 3 hours. It was then transferred into fresh DIG Easy Hyb buffer with 100 µg/ml salmon sperm DNA (Thermo) and 1 kb DIG labelled probe (Fratta *et al*, 2014) and left overnight at 46 ºC. The membrane was washed for 15 minutes in 0.5X SSC with 0.1% SDS at a rising temperature from 46ºC to 65ºC, before being washed twice more at 65ºC. Following washes, the membrane was processed using the Roche DIG wash and block buffer set following manufacturer's instructions and using the AP-conjugated anti-digoxigenin (11093274910, Roche) at 1:10,000. The membrane was incubated in CDP-star ready to use (Roche) sealed in acetate and exposed to film (Roche Lumi-Film chemiluminescent detection film) for >1 hour before being developed using an X4 automatic processor (XOgraph).

Immunofluorescence staining of iPSC-neurons

iPSC-neurons were grown on laminin coated coverslips and were fixed in 4% paraformaldehyde in PBS for 15 minutes then washed three times in PBS supplemented with 0.3% Triton-X (PBST) and blocked with 5% BSA in PBST for 30 minutes. Cells were incubated overnight at 4 °C with primary antibody (ChAT 1:100 Millipore AB144P and TUJ1 1:1000 Biolegend 802001) in 5% BSA in PBST. Cells were washed in PBST three times then incubated with complementary Alexa Fluor secondary antibody (1:500) dilution for one hour at room temperature. Cells were washed once in PBST containing DAPI for ten minutes then twice more in PBST. Coverslips were mounted onto glass slides in Dako mounting medium. Images were acquired using an LSM700 confocal microscope (Zeiss) using a plan-apochromat 40x/1.4 NA oil immersion objective.

XTT assay in human fibroblasts

WI-38 non-transformed lung fibroblasts were seeded at a density of 6,000 cells/well in 96 well-plates in Eagle`s Minimum Essential Medium supplemented with 10% FBS, 1% non-essential amino acids and 1% L-glutamine and maintained at 37°C with 5% CO_2. 24 hrs later, fibroblasts were treated with 0.04, 0.1, 0.2, 0.4, 1, 2, 4, 10, 20 and 40 µM of DB1246, DB1247, DB1273 or cisplatin as a positive control with eight replicates. Cell viability was measured after 4 days using the XTT assay kit II (Roche) according to the manufacturer instructions.

***Drosophila* larvae quantitative RT-PCR**

Total RNA was extracted from 5 larvae for each replicate using Trizol (Invitrogen) according to the manufacturer's instructions and treated with DNAse I (Roche). cDNA was synthesized using 1 µg of total RNA for all samples, with a SuperScript III first strand cDNA synthesis kit (Invitrogen) and an equimolar mixture of oligo dT and random hexamer primers. Real-time qRT–PCR was carried out using Power SYBR Green Master Mix (Applied Biosystems) with previously described primers for the pUASTattB transgene (Tran *et al*, 2015) and normalised to α-tubulin. The amplified transcripts were quantified using the comparative Ct method and presented as normalized fold expression (ΔΔCt). Oligonucleotide sequences are provided in Table S2.

***Drosophila* confocal imaging**

To investigate bioavailability of DB1273, daGAL4>36R first instar larvae were treated with vehicle, or 1 mM of DB1273, and dissected after 5-6 days at the third instar stage. 2-3 larvae were dissected per condition. Larval tissues were dissected in PBS and then fixed in 4 % PFA in PBS at room temperature for 20 mins. Larval tissues were washed in PBS and whole

mounted onto glass slides in VECTASHIELD® mounting medium with DAPI (Vector Laboratories, H-1200). Z-stack images of larval tissues were acquired using an LSM710 confocal microscope (Zeiss) with a plan-apochromat 40x/1.4 NA oil immersion objective. Tissues were excited using the 488 nm laser excitation to detect DB1273. DB1273 fluorescence intensity was set to peak for individual larval tissues, using the same imaging settings for 0 mM and 1 mM treatments for each tissue.

Appendix supplementary figures

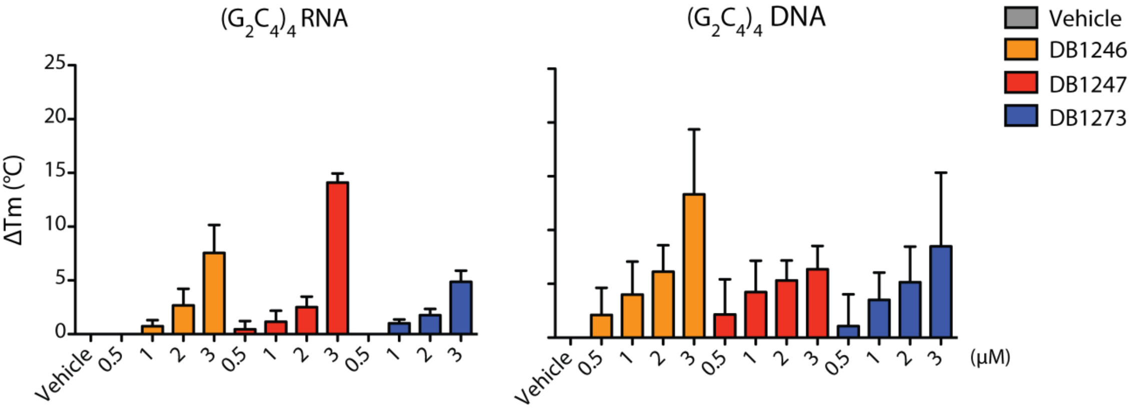

Appendix Fig. S1. Dose-response of small molecule binding to antisense GGCCCC repeats. A FRET assay was used to measure the difference in melting temperature (ΔT_m) of $(G_2C_4)_4$ RNA or DNA G-quadruplexes in the presence of 0 – 3 µM DB1246, DB1247 and DB1273, compared to vehicle (DMSO). Data is shown as mean ± s.d., n=3 independent experiments.

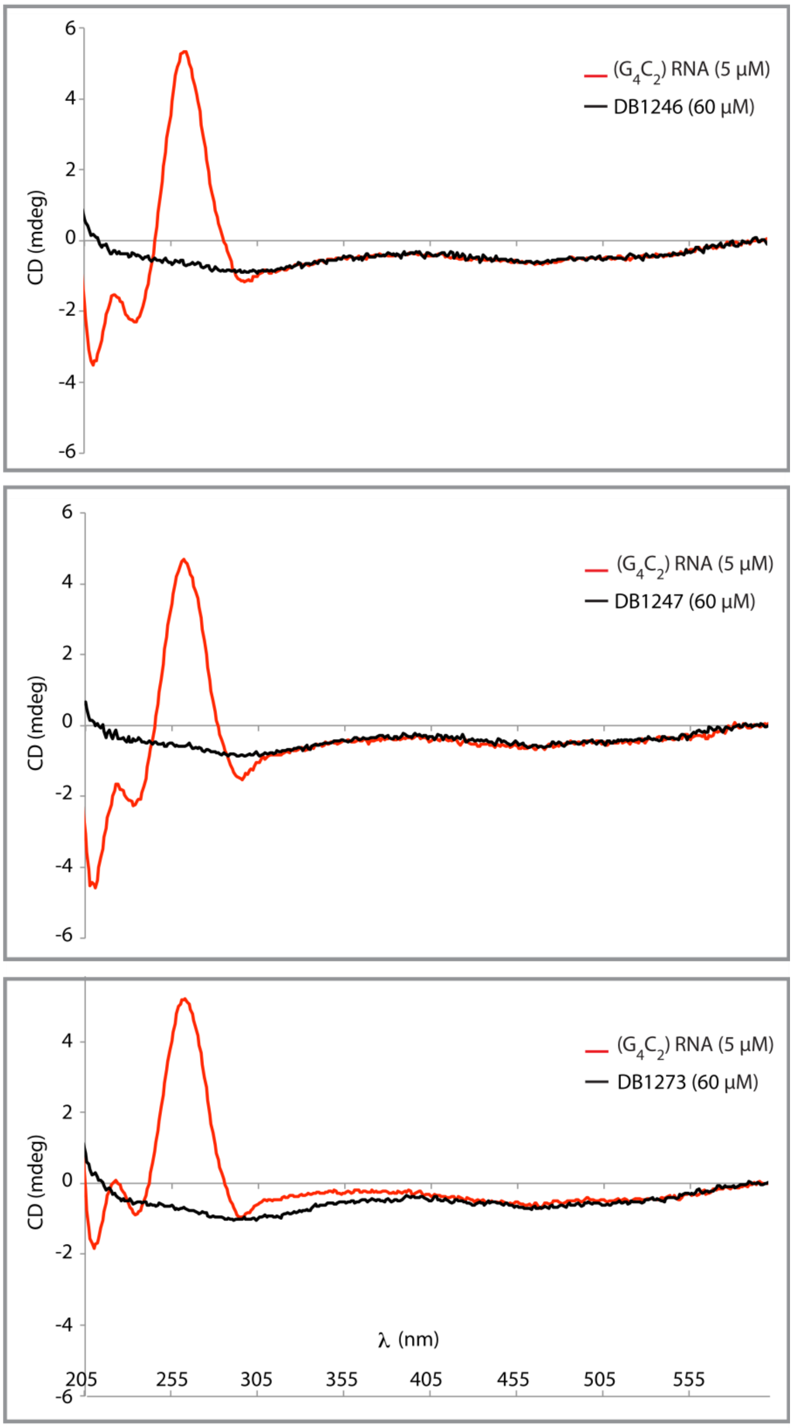

Appendix Fig. S2. Small molecules alone give no CD signal. $(G_4C_2)_4$ RNA alone (red line) gives a characteristic CD spectrum, whereas DB1246, DB1247 and DB1273 alone (black lines) give no signal, confirming that the induced CD signal when RNA and small molecule are combined (Figure 1D) is due to a specific small molecule-RNA interaction.

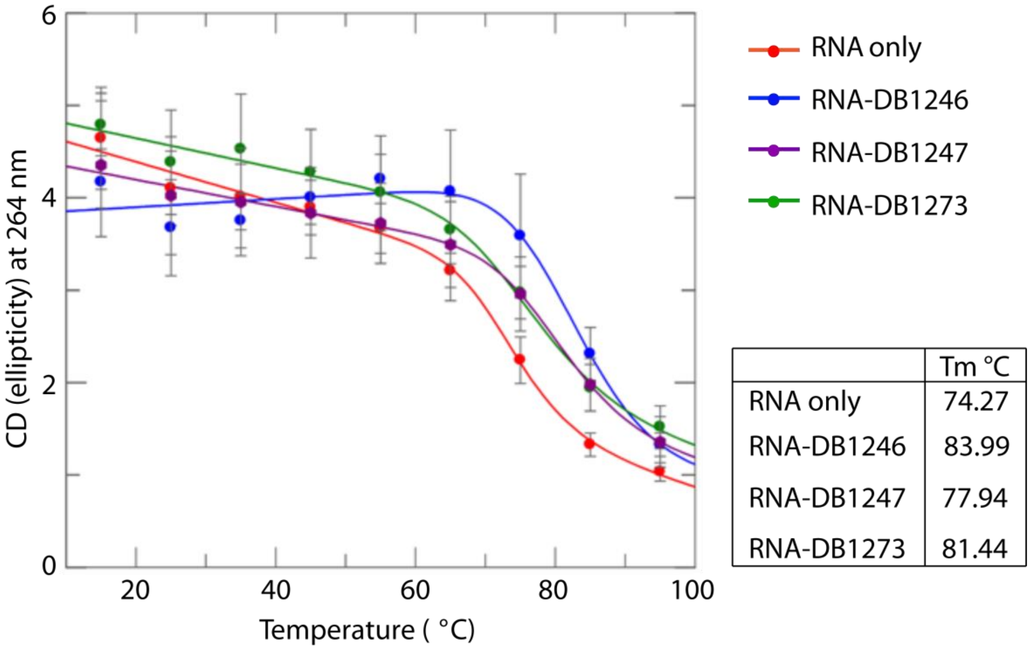

	Tm °C
RNA only	74.27
RNA-DB1246	83.99
RNA-DB1247	77.94
RNA-DB1273	81.44

Appendix Fig. S3. $(G_4C_2)_4$ RNA CD denaturation curves in the presence of each small molecule. CD spectra were measured during a thermal unfolding, with increasing temperature from 15 to 95 °C in steps of 10 °C. All three compounds increase the melting temperature (T_m) of the $(G_4C_2)_4$ RNA. Values represent mean ± s.d. (n=3 independent experiments). T_m was calculated using the van't Hoff equation.

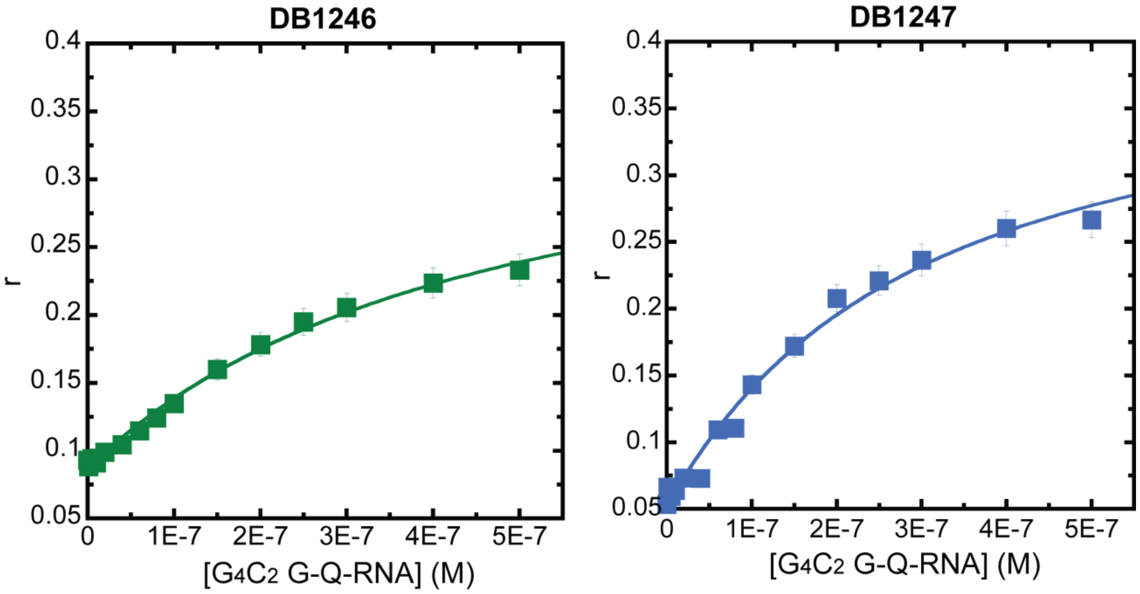

Appendix Fig. S4. Fluorescence anisotropy titration of DB1246 and DB1247 to G_4C_2 G-Q RNA. Change in anisotropy value r were plotted against the concentration of G_4C_2 G-Q RNA. Compound concentrations were held near 50 nM in 10 mM Tris-HCl, 50 mM KCl in nuclease-free water (pH 7.4) with increasing concentration of RNA to obtain K_D values for 410 nM ± 79 258 ± 60 nM for DB1246 and DB1247 respectively, using a 1 to 1 binding model as described in the methods section.

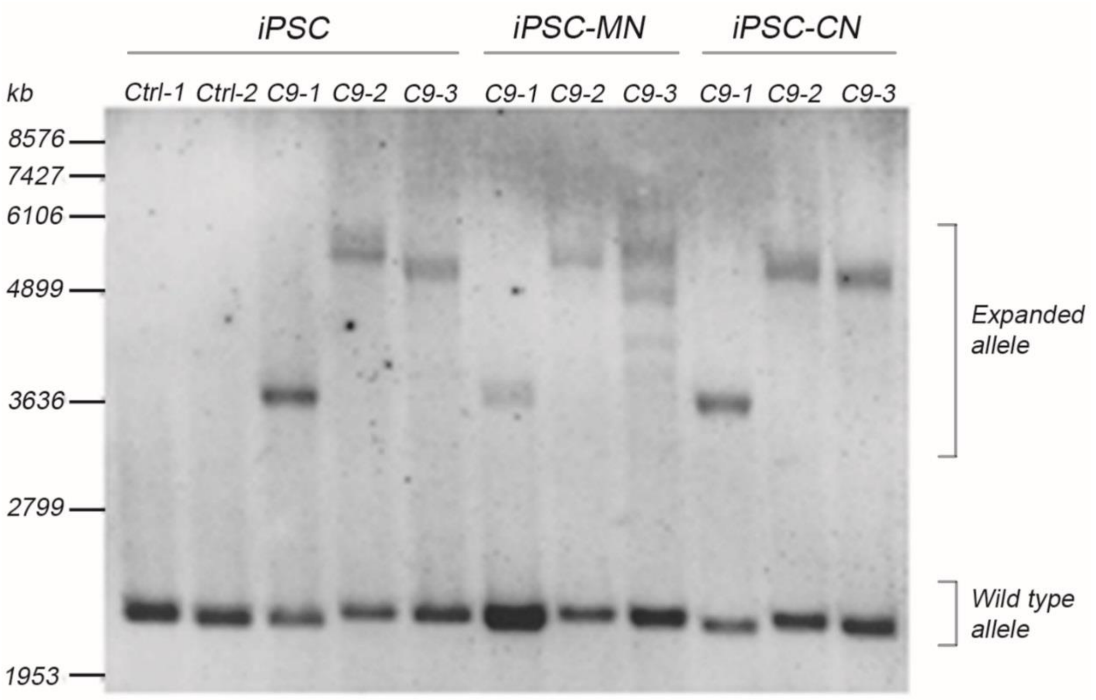

Appendix Fig. S5. Expanded G_4C_2 repeats in *C9orf72* patient lines and derived motor and cortical neurons. Southern blotting with a *C9orf72*-specific probe reveals expansions in the *C9orf72* patient iPSC lines (C9-1, -2, -3) that are not present in control lines (Ctrl-1, -2) and that remain largely stable following differentiation into either motor neurons (MN) or cortical neurons (CN).

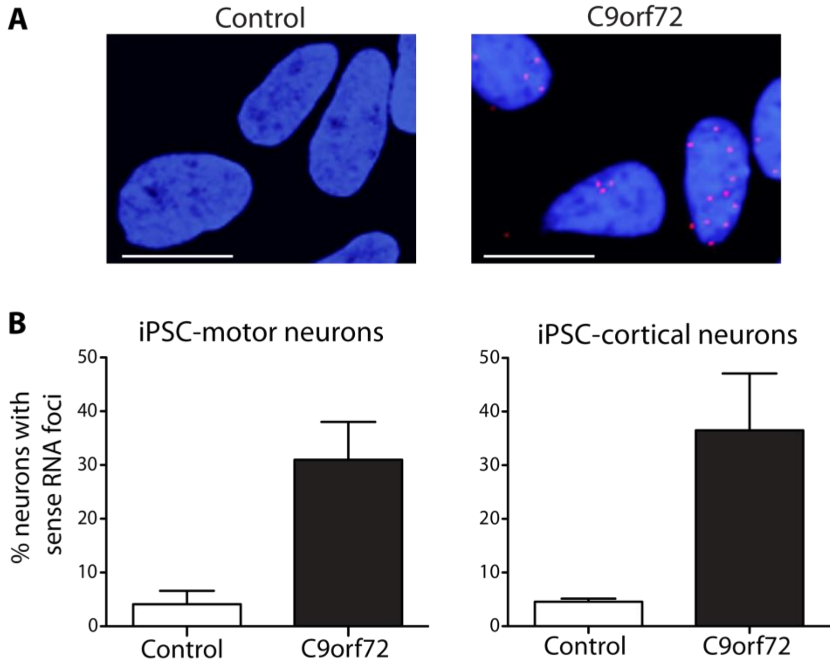

Appendix Fig. S6. RNA foci are specific to *C9orf72* patient iPSC-cortical and motor neurons. G_4C_2 repeat RNA foci were detected by FISH and automatically quantified using image analysis software (Volocity, Perkin Elmer). (**A**) Representative image of RNA foci (red) within iPSC-cortical neurons; nuclei are visualised with DAPI (blue). Scale bars represent 10 µm. (**B**) Quantification shows RNA foci are specific to *C9orf72* patient lines. Some background fluorescence cannot be distinguished from RNA foci by the automated software, leading to low levels of non-specific signal in controls. Two independent control iPSC lines and three independent *C9orf72* lines were analysed, with 1-3 independent differentiations per line and >70 cells quantified per line. Bars show mean and SEM.

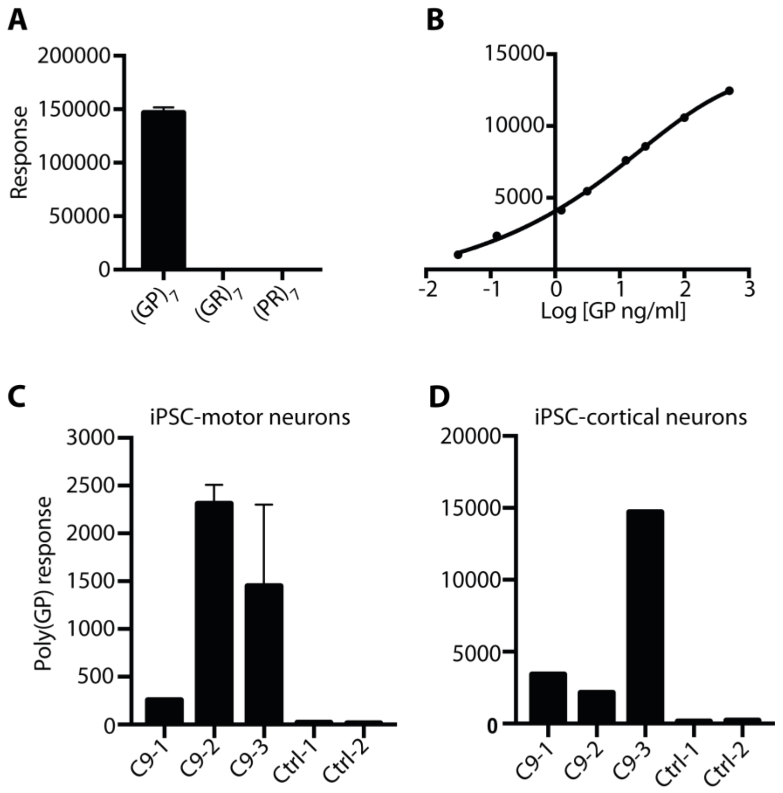

Appendix Fig. S7. Poly(GP) is specifically detected in *C9orf72* patient iPSC-cortical and -motor neurons. A MesoScale Discovery (MSD) immunoassay was developed to detect poly(GP). (**A**) The MSD immunoassay specifically detects $(GP)_7$ peptide but not other dipeptide repeat proteins $(GR)_7$ or $(PR)_7$. (**B**) Representative standard curve of MSD response at different concentrations of $(GP)_7$ peptide calibrator, R^2=0.99. Lower limit of detection is 0.034ng/ml. Poly(GP) is detected by MSD immunoassay in both *C9orf72* (C9) patient iPSC-motor neurons (**C**) and iPSC-cortical neurons (**D**) and not in control (Ctrl) lines. Data in (C) are the average and SEM of 3 inductions per line (3 independent C9 lines and 2 Ctrl lines). Data in (D) is the average response of two technical replicates for one induction of the same lines as in (C).

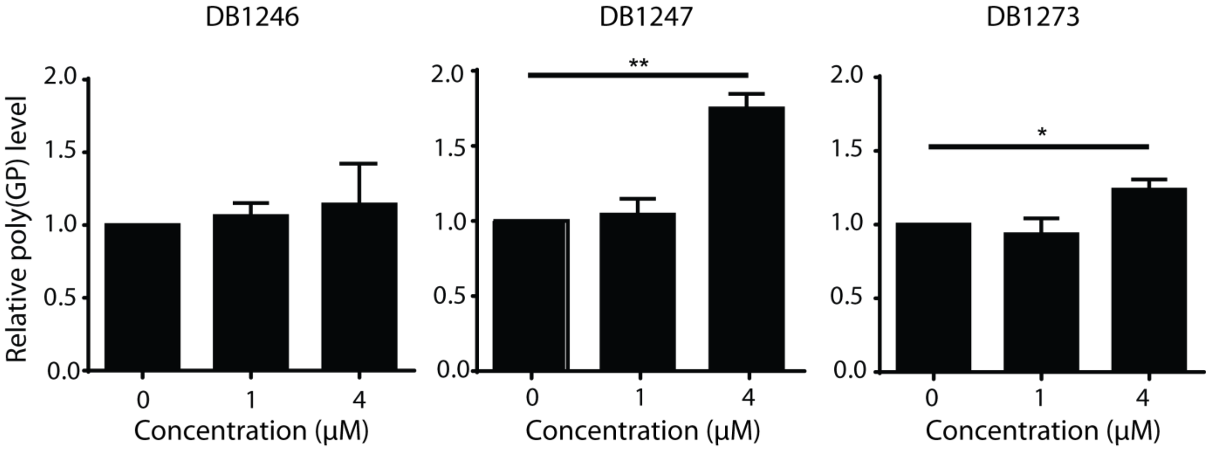

Appendix Fig. S8. GGGGCC repeat G-quadruplex binding small molecules at 1 or 4 µM doses do not reduce poly(GP) in patient iPSC-motor neurons. Poly(GP) was detected by MSD immunoassay in *C9orf72* patient iPSC-motor neurons treated for 4 days with 1 or 4 µM of DB1246, DB1247 and DB1273. Relative to those treated with DMSO as a control there is no reduction in poly(GP) in iPSC-motor neurons after treatment with the three compounds. Treatments on 3 independent *C9orf72* iPSC lines with 1-6 differentiations per line. * $p<0.05$, ** $p<0.01$, one sample two-tailed t-test versus normalised control. $p=0.0044$ (DB1247 4 µM), $p=0.0413$ (DB1273 4 µM).

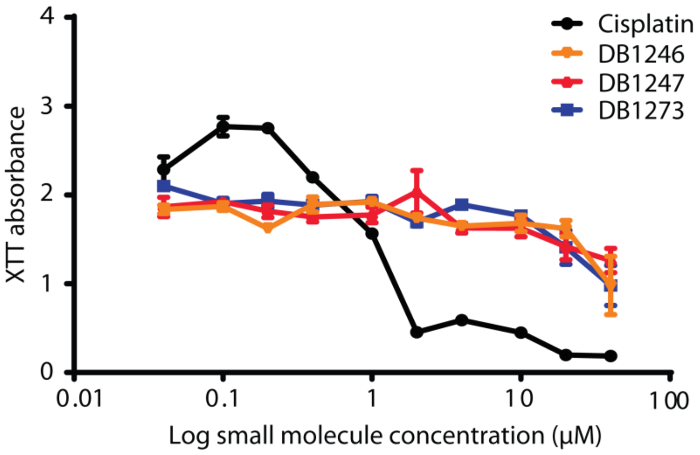

Appendix Fig. S9. Small molecules have low toxicity in human fibroblasts. XTT cell death assay of control human fibroblasts treated for 4 days with G_4C_2 repeat G-quadruplex binding small molecules or cisplatin as a positive control at a concentration range of 0-40 µM. n=3 independent experiments for all data points except cisplatin (n=2). Data are shown as mean and SEM.

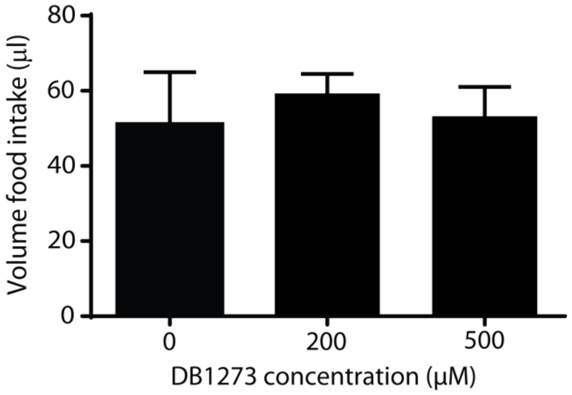

Appendix Fig. S10. Food intake is not altered in adult *Drosophila* fed G_4C_2 repeat G-quadruplex binding small molecules. DaGS>36R adult *Drosophila* were fed RU486 to induce expression of the GGGGCC repeats and in addition were treated for 7 days with vehicle, 200 µM or 500 µM DB1273 in liquid food via capillaries. The total volume of food intake over the 7 day treatment, per vial of 5 flies, is shown and was no different across flies treated with different concentrations of DB1273. Data are shown as the mean and SEM of 3 experiments. One way ANOVA with Dunnett's post hoc test, $p>0.05$. Genotype was w; UAS-(GGGGCC)36/daGS (daGS>36R).

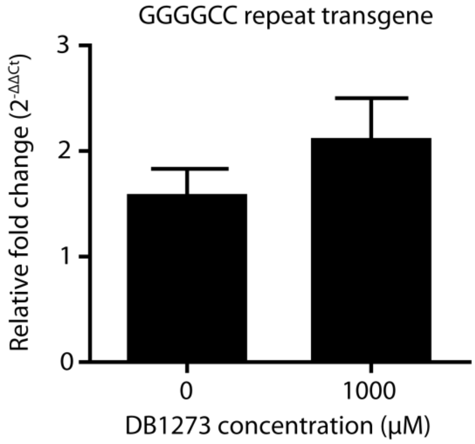

Appendix Fig. S11. Larval DB1273 treatment does not affect expression levels of the $(G_4C_2)_{36}$ transgene. DaGAL4>36R *Drosophila* first instar larvae were treated for 5 days, until they reached the L3 stage, with vehicle or 1 mM DB1273. Quantitative real-time PCR was performed to measure the expression level of the $(G_4C_2)_{36}$ transgene, by amplification of the downstream pUAST vector sequence and normalized to α-tubulin. No significant difference was observed (two-tailed t test), n=4 per group, with 5 larvae per replicate. Data are shown as mean and SEM. Genotype was w; UAS-(GGGGCC)36/+; daGAL4/+ (daGAL4>36R).

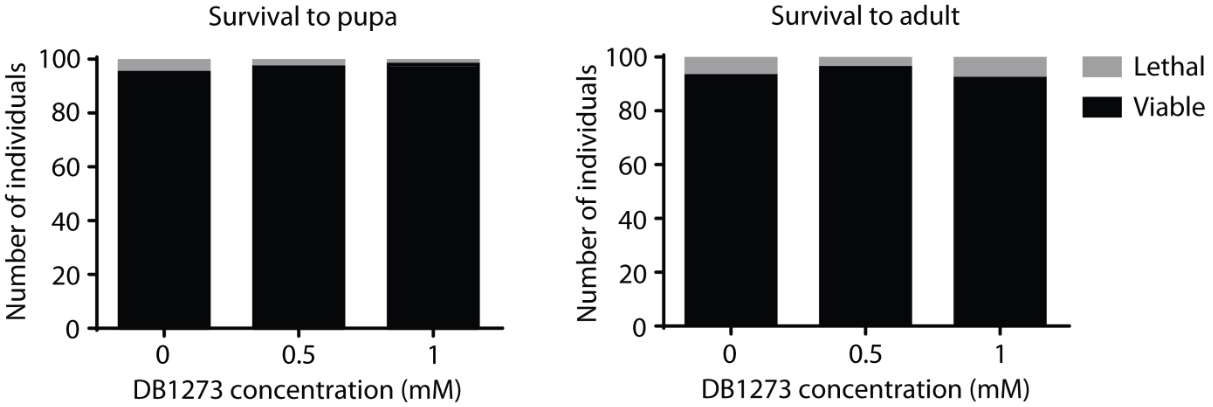

Appendix Fig. S12. The G_4C_2 repeat G-Q binding small molecule, DB1273, is not toxic in *Drosophila*. Control daGAL4/+ first instar (L1) larvae treated were with vehicle, 0.5 mM or 1 mM of the G_4C_2 repeat G-Q binding small molecule DB1273. The numbers reaching the pupal and adult stage of development were counted. Treatment with DB1273 does not affect survival to the pupal or adult stage of development, after 7 days and 10 days respectively. Data are shown as proportion reaching each stage of development out of 100 L1 larvae per condition. No significant differences in survival were observed, Chi squared test, $p>0.05$. Genotype was w; +; daGAL4/+.

ID	Gender	Diagnosis (at the time of biopsy)	Age at disease onset (years)	Age at biopsy (years)	*C9orf72* G_4C_2 repeat size
C9-1	M	ALS	39	50	~241
C9-2	M	ALS	52	58	~638
C9-3	M	ALS	72	74	~602
Ctrl1	M	N/A	N/A	64	N/A
Ctrl 2	M	N/A	N/A	78	N/A

Appendix Table S1. iPSC lines used in this study.

Name	Sequence
TBP-F	ACGCCGAATATAATCCCAAG
TBP-R	CTTCACTCTTGGCTCCTGTG
GAPDH-F	ACTAACCCTGCGCTCCTG
GAPDH-R	CCCAATACGACCAAATCAGA
MCM2-F	CGAGATAGAGCTGACTGGCA
MCM2-R	GCTAGGATGACAGTGGCAAA
V1-F	GCGGTGGCGAGTGGATAT
V1+3R	TGGGCAAAGAGTCGACATCA
V3-F	GAGCAGGTGTGGGTTTAGGAGA
V2-F	TCATCTATGAAATCACACAGTGTTC
V2-R	GGTATCTGCTTCATCCAGCTT
LNA PrimeTime Probes	
LNA V1+V3	5' 6-FAM/ATTTGGATAATGTGACAGTTGG/ 3' IBFQ
LNA V2	5' 6-FAM/ATGATGATGATATTGGTGAC/ 3' IBFQ
pUASTattb-F	TTCCAACCTATGGAACTGATGA
pUASTattb-R	GGTTTTCCTCATTAAAGGCATTC
α-tubulin-F	TGGGCCCGTCTGGACCACAA
α-tubulin-R	TCGCCGTCACCGGAGTCCAT

Appendix Table S2. Oligonucleotides used for quantitative RT-PCR.

Appendix supplementary references

Fratta P, Polke JM, Newcombe J, Mizielinska S, Lashley T, Poulter M, Beck J, Preza E, Devoy A, Sidle K, Howard R, Malaspina A, Orrell RW, Clarke J, Lu CH, Mok K, Collins T, Shoaii M, Nanji T, Wray S et al (2014) Screening a UK amyotrophic lateral sclerosis cohort provides evidence of multiple origins of the C9orf72 expansion. *Neurobiol Aging* 36: 546.e1-e7

Lea WA, Simeonov A (2011) Fluorescence Polarization Assays in Small Molecule Screening. *Expert opinion on drug discovery*, 6: 17-32.

Roehrl MH, Wang JY, Wagner G (2004) A general framework for development and data analysis of competitive high-throughput screens for small-molecule inhibitors of protein-protein interactions by fluorescence polarization. *Biochemistry* 43: 16056-16066

Tran H, Almeida S, Moore J, Gendron TF, Chalasani U, Lu Y, Du X, Nickerson JA, Petrucelli L, Weng Z, Gao FB (2015) Differential Toxicity of Nuclear RNA Foci versus Dipeptide Repeat Proteins in a Drosophila Model of C9ORF72 FTD/ALS. *Neuron* 87: 1207-1214
