# Supplementary material for: G‐quadruplex‐binding small molecules ameliorate C9orf72 FTD/ALS pathology in vitro and in vivo
Source: EMBO Mol Med. 2017 Nov 7;10(1):22–31. doi: 10.15252/emmm.201707850 (PMC5760849; doi:10.15252/emmm.201707850)
Supplement: Supplementary file 2 — Expanded View Figures PDF [file EMMM-10-22-s002.pdf]

Expanded View Figures

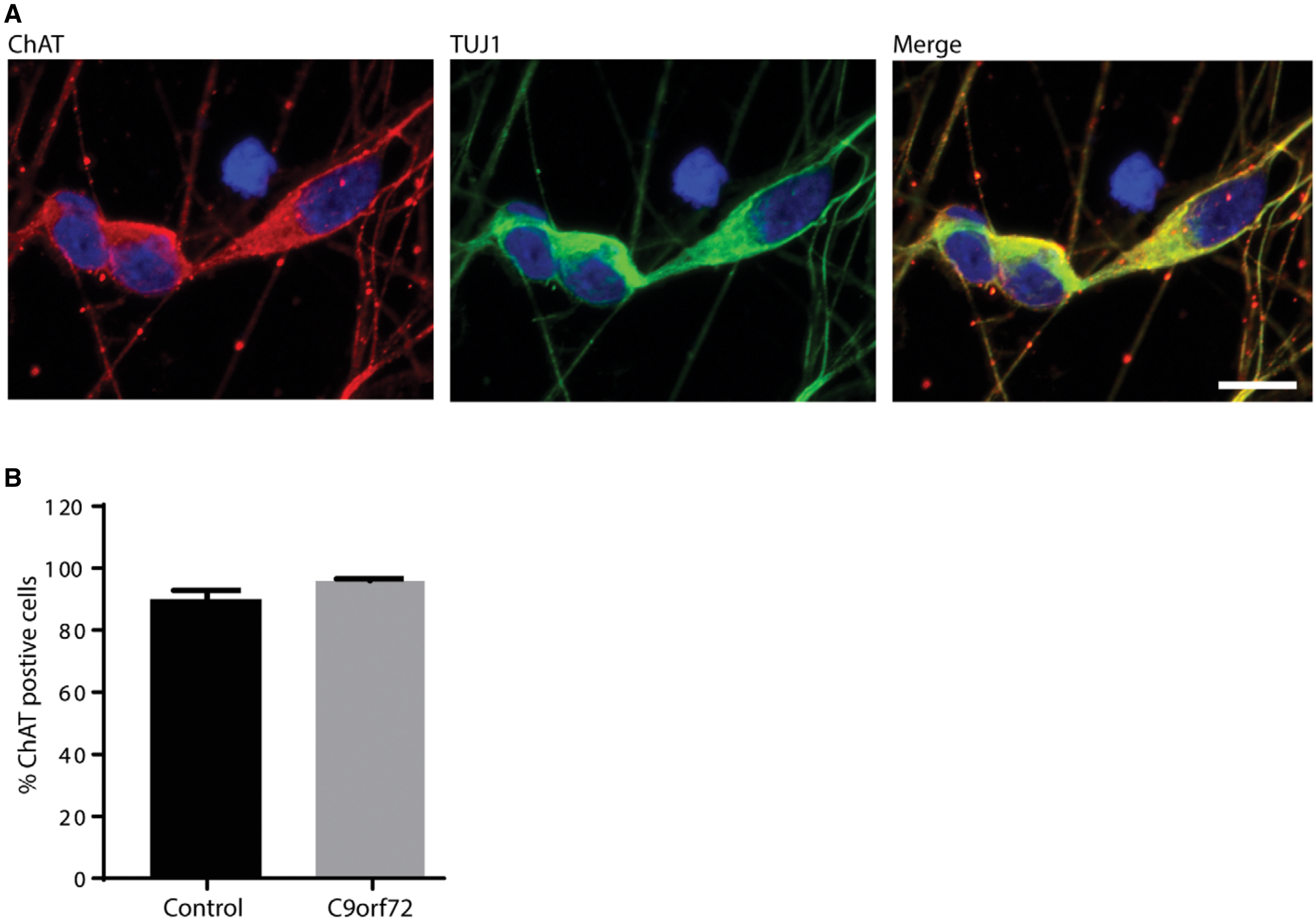

Figure EV1. Highly efficient differentiation of iPSCs into motor neurons is not affected by *C9orf72* repeat expansion.

A *C9orf72* iPSC-motor neurons express choline acetyltransferase (ChAT) and beta-tubulin (TUJ1). Scale bar represents 10 μm.

B The percentage of total cells positive for ChAT was quantified after differentiation into motor neurons. No difference was observed between control and *C9orf72*-derived iPSC-motor neurons, with approximately 90% of all cells converted to ChAT-positive motor neurons. Two independent control iPSC lines and three independent *C9orf72* lines were analysed, with one to two independent differentiations per line and > 100 cells quantified per line. Bars show the average and SEM. $P > 0.05$, Mann–Whitney *U*-test.

© 2017 The Authors

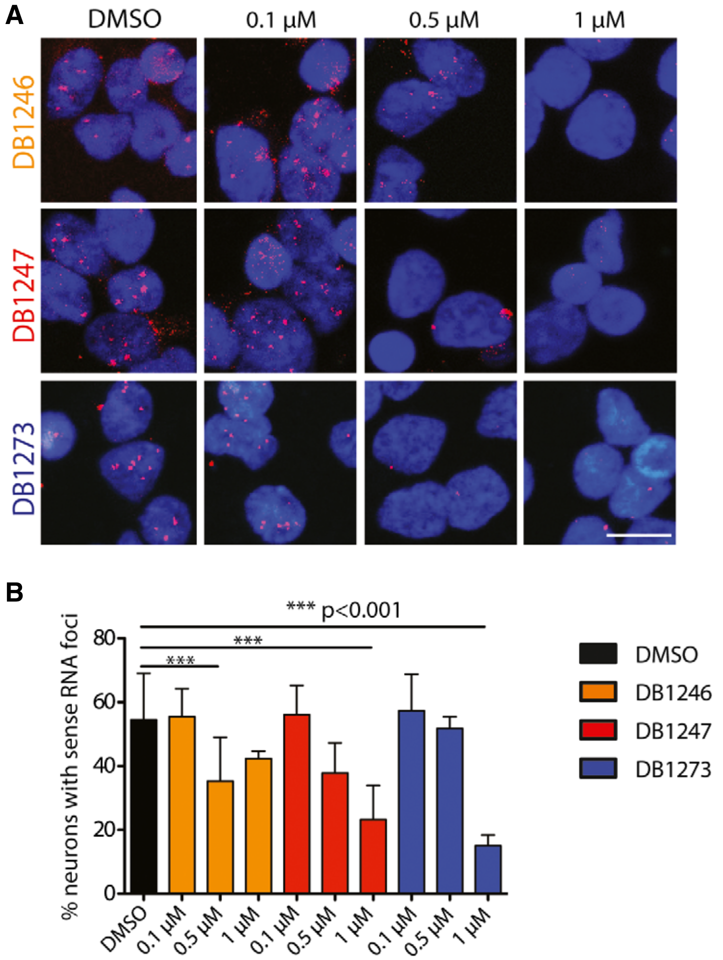

Figure EV2. G_4C_2 repeat G-quadruplex binding small molecules reduce RNA foci in *C9orf72* patient iPSC-cortical neurons.

G_4C_2 repeat RNA foci were detected by FISH and automatically quantified using image analysis software (Volocity, PerkinElmer).

A Representative images of RNA foci (red) within iPSC-cortical neurons; nuclei are visualised with DAPI (blue). Scale bar represents 10 µm.

B Quantification shows RNA foci are significantly reduced by all three small molecules, DB1246, DB1247 and DB1273, at a concentration of 1 µM for 4 days. Data are shown as the average and SD of the percentage of neurons containing RNA foci in 5–10 40× fields of view for one *C9orf72* patient iPSC-cortical neuron line. ****P* = 0.0006 (DB1246, 0.5 µM), ****P* = 0.0001 (DB1247, 1 µM), ****P* = 0.0001 (DB1273, 1 µM), one-way ANOVA with Dunnett's *post hoc* test versus DMSO.

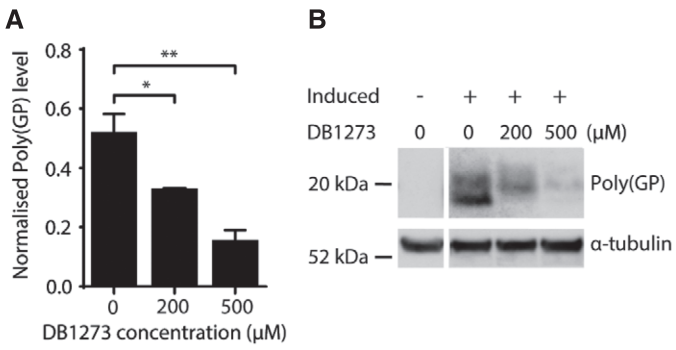

Figure EV3. G_4C_2 repeat G-quadruplex binding small molecules reduce poly(GP) in GGGGCC repeat-expressing *Drosophila*.

A Treatment of adult *Drosophila* ubiquitously expressing 36 G_4C_2 repeats for 7 days with DB1273 leads to a significant reduction in poly(GP) levels (normalised to α-tubulin) relative to vehicle-treated controls. Data are shown as the mean and SEM of three experiments. **P* = 0.0463 (200 µM versus 0 µM), ***P* = 0.0026 (500 µM versus 0 µM), one-way ANOVA with Dunnett's *post hoc* test. Genotype was *w1118; daGS/UAS-36(GGGGCC) (daGS>36R)*.

B Representative poly(GP) immunoblot. Poly(GP) appears as a doublet in the induced *daGS>36R* flies and is not present in uninduced flies, confirming its generation is due to expression of the $(G_4C_2)36$ repeats. Genotype was *w1118; daGS/UAS-36(GGGGCC) (daGS>36R)*.

© 2017 The Authors

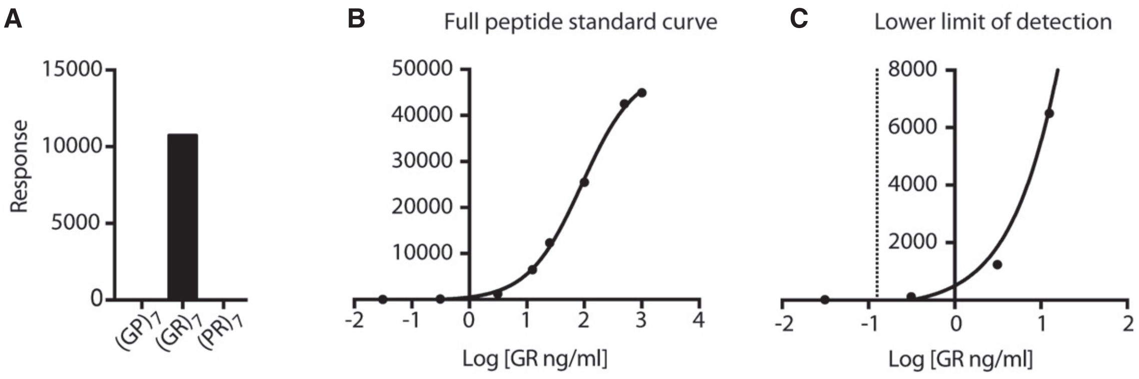

Figure EV4. Poly(GR) MSD immunoassay validation. A MesoScale Discovery (MSD) immunoassay was developed to detect poly(GR).

A The MSD immunoassay specifically detects $(GR)_7$ peptide but not other dipeptide repeat proteins $(GP)_7$ or $(PR)_7$.

B Representative standard curve of MSD response at different concentrations of $(GR)_7$ peptide calibrator, $R^2 = 0.99$.

C Enhanced view of (B) to show the lower limit of detection of the assay, which is 0.125 ng/ml (dashed line).

© 2017 The Authors

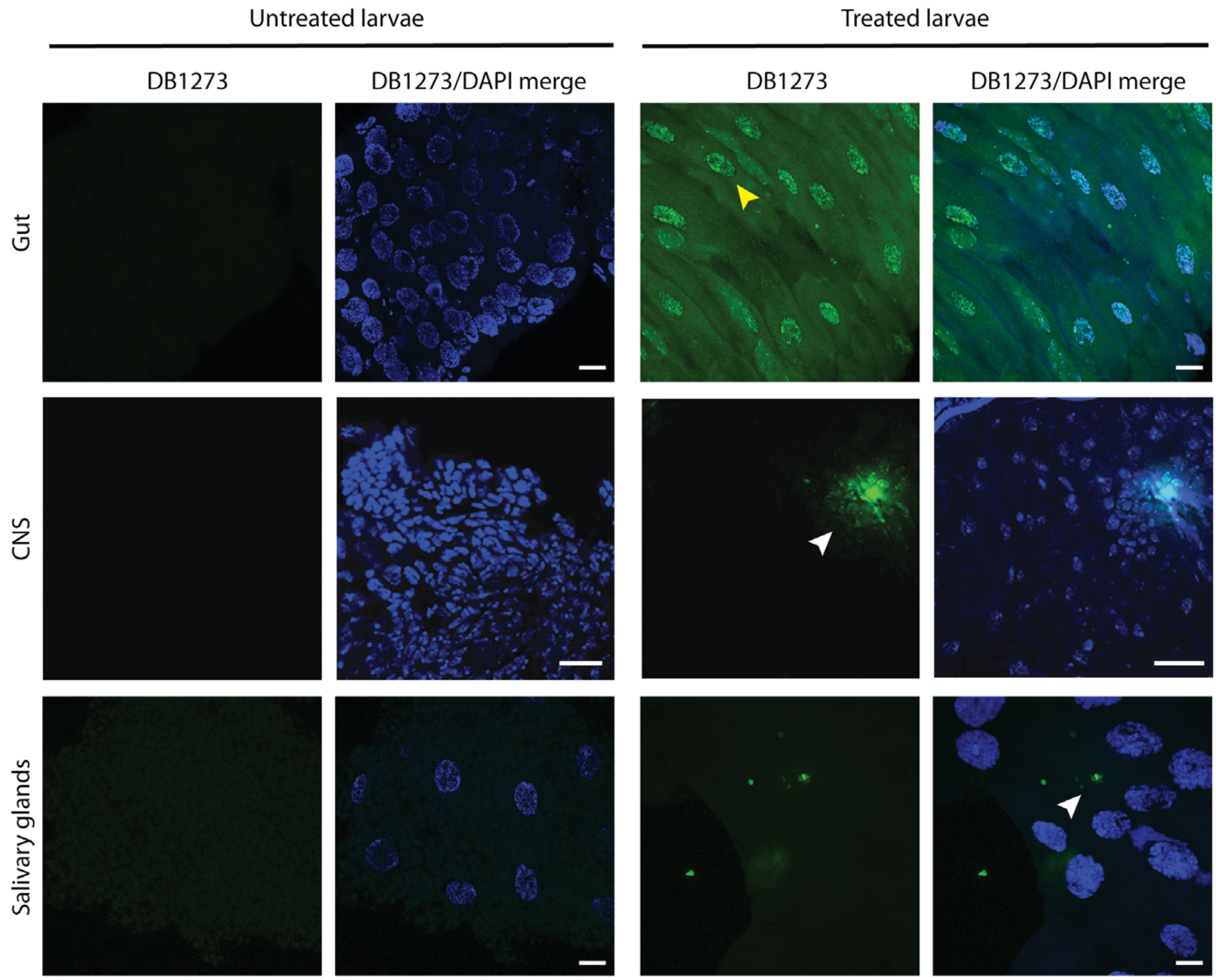

Figure EV5. The distribution of the G_4C_2 repeat G-Q-binding small molecule, DB1273, in *Drosophila* larvae.

daGAL4>36R first-instar larvae were treated with vehicle, or 1 mM of the G_4C_2 repeat G-Q-binding small molecule, DB1273, and dissected after 5–6 days at the third-instar stage. Representative images of *Drosophila* tissues imaged using confocal microscopy using a 488-nm laser excitation to detect DB1273, and with DAPI nuclear stain (blue). DB1273 is detected in treated larvae in gut epithelial cells within nuclei (yellow arrowhead) and the cytoplasm. Infrequent fluorescent puncta are detected in other larval tissues, in the central nervous system (CNS) and salivary glands (white arrowheads). Scale bar: 20 µm. Genotype was *ω118; UAS-36(GGGGCC)/+; daGAL4/+* (*daGAL4>36R*).

 © 2017 The Authors
